# Perspectives of pediatric oncologists and palliative care physicians on the therapeutic use of cannabis in children with cancer

**DOI:** 10.1002/cnr2.1551

**Published:** 2021-10-21

**Authors:** Sapna Oberoi, Jennifer L. P. Protudjer, Adam Rapoport, Shahrad R. Rassekh, Bruce Crooks, Harold Siden, Kathleen Decker, Prasanna Ananth, Stacy Chapman, Lynda G. Balneaves, Magimairajan Issai Vanan, Lauren E. Kelly

**Affiliations:** ^1^ Department of Pediatrics and Child Health University of Manitoba Winnipeg Canada; ^2^ Department of Pediatric Hematology‐Oncology CancerCare Manitoba Winnipeg Canada; ^3^ Children's Hospital Research Institute of Manitoba Winnipeg Manitoba Canada; ^4^ George and Fay Yee Centre for Healthcare Innovation Winnipeg Canada; ^5^ Department Foods and Human Nutritional Sciences University of Manitoba Winnipeg Canada; ^6^ Paediatric Advanced Care Team Hospital for Sick Children Toronto Ontario Canada; ^7^ Emily's House Children's Hospice Toronto Ontario Canada; ^8^ Department of Paediatrics Faculty of Medicine, University of Toronto Toronto Ontario Canada; ^9^ Department of Family & Community Medicine Faculty of Medicine, University of Toronto Toronto Ontario Canada; ^10^ Division of Oncology/Hematology/BMT, Department of Pediatrics University of British Columbia Vancouver Canada; ^11^ Pediatric Hematology/Oncology & Medical Education IWK Health Centre Halifax Canada; ^12^ Department of Pediatrics, Faculty of Medicine University of British Columbia Vancouver Canada; ^13^ Department of Community Health Sciences University of Manitoba Winnipeg Canada; ^14^ Section of Pediatric Hematology/Oncology, Department of Pediatrics Yale School of Medicine; ^15^ College of Nursing University of Manitoba Winnipeg Canada

**Keywords:** cancer, cannabidiol, cannabis, pediatrics, symptom control, tetrahydrocannabinol

## Abstract

**Background:**

Children with cancer are increasingly using cannabis therapeutically.

**Aim:**

The purpose of this study was to determine the perspectives and practices of pediatric oncologists and palliative care physicians regarding the use of cannabis for medical purposes among children with cancer.

**Methods:**

A self‐administered, voluntary, cross‐sectional, deidentified online survey was sent to all pediatric oncologists and palliative care physicians in Canada between June and August 2020. Survey domains included education, knowledge, and concerns about cannabis, views on its effectiveness, and the importance of cannabis‐related research. Data were analyzed using descriptive statistics.

**Results:**

In total, 122/259 (47.1%) physicians completed the survey. Although 62.2% of the physicians completed some form of training about medical cannabis, nearly all (95.8%) desired to know more about the dosing, side effects, and safety of cannabis. Physicians identified a potential role of cannabis in the management of nausea and vomiting (85.7%), chronic pain (72.3%), cachexia/poor appetite (67.2%), and anxiety or depression (42.9%). Only four (0.3%) physicians recognized cannabis to be potentially useful as an anticancer agent. Nearly all physicians reported that cannabis‐related research for symptom relief is essential (91.5%) in pediatric oncology, whereas 51.7% expressed that future studies are necessary to determine the anticancer effects of cannabis.

**Conclusions:**

Our findings indicate that most pediatric oncologists and palliative care physicians recognize a potential role for cannabis in symptom control in children with cancer. Well‐conducted studies are required to create evidence for cannabis use and promote shared decision making with pediatric oncology patients and their caregivers.

## 1 INTRODUCTION

Cannabis products are derived from the plant *Cannabis Sativa* and are available in many forms, including dried flowers; hash; extracts, such as oil and shatter; and edibles.^1^, ^2^ Cannabis plant contains more than 100 compounds called phytocannabinoids, many of which are bioactive.^2^ The two most prominent cannabinoids are Δ9‐tetrahydrocannabinol (THC) and cannabidiol (CBD).^3^ Recent changes in cannabis legislation in Canada have made cannabis products more accessible and have raised public interest in using cannabis for medical purposes, including for children with cancer.^4^ Outside of intractable epilepsy, minimal data exist on the therapeutic use of cannabis in children, despite its widespread use and interest. Several clinical trials have examined the therapeutic role of CBD for the treatment of seizures in children with Dravet syndrome, Lennox–Gastaut syndrome or tuberous sclerosis.^5^, ^6^, ^7^, ^8^ Overall, these trials demonstrated that CBD significantly reduced the frequency of seizures compared to placebo.^5^, ^6^, ^8^ The majority of the adverse effects associated with CBD use were non‐severe and included diarrhea, vomiting, fatigue, pyrexia, somnolence, and decreased appetite.^5^, ^6^, ^8^

However, limited data exist on the use of cannabis for conditions outside of epilepsy. Despite limited evidence on the safety or efficacy of cannabis in pediatric oncology, parents or caregivers of children with cancer may use cannabis products for various indications, including nausea, vomiting, pain, lack of appetite, mood, and as an anticancer agent.^9^, ^10^, ^11^ In a few recently published studies, children and young adults with cancer reported using cannabis products for nausea and vomiting, depressed mood, sleep disturbances, pain, poor appetite and weight loss.^9^, ^10^ In a study from Israel, nearly 80% of children and their parents, believed that cannabis alleviated their physical and psychological distress; however, the study used no control group, and outcomes were self‐reported.^9^

Pediatric oncologists are increasingly facing questions from patients and their caregivers regarding the role of cannabis for symptom control and treatment of cancer. Lack of evidence, concern for adverse psychiatric and cognitive effects, and dosing guidelines have generally limited physician support for using cannabis products in children.^12^, ^13^, ^14^ In a recently conducted survey of interdisciplinary providers in pediatric oncology in the United States, 30% of providers received ≥1 request for cannabis in the previous month.^12^ A nationwide survey of medical oncologists from the United States showed that despite insufficient information on cannabis, 80% of oncologists discussed and 46% recommended cannabis to their adult patients with cancer.^15^ The results of these surveys, however, may not be directly applicable to the Canadian setting due to differences in the healthcare systems, medical cannabis access, and recent legalization of recreational cannabis throughout Canada.^16^, ^17^

Therefore, to understand perspectives concerning the therapeutic use of cannabis in Canadian children with cancer and determine the necessity for cannabis‐related research, we surveyed pediatric oncologists and palliative care physicians in Canada.

## 2 MATERIALS AND METHODS

We conducted a cross‐sectional study using a self‐administered, deidentified online survey. Pediatric oncologists, palliative care physicians, and fellows in both sub‐specialties were eligible to participate in this study if they provided clinical care to oncology patients at a Canadian pediatric academic health sciences center. We convened a content expert panel of pediatric oncologists, pediatric palliative care physicians, pharmacologists, and researchers to develop this survey. The specific survey domains were informed by discussion and consensus among the members of the expert panel. Survey domains included education and desired knowledge about medical cannabis, perspectives on the use of medical cannabis in pediatric oncology, concerns about the use of medical cannabis, and attitudes toward future research. We generated survey questions in each domain by reviewing published survey instruments^12^, ^15^ and discussing with the content expert panel. The survey was piloted with five pediatric oncologists and palliative care physicians to test flow, salience, appropriateness, ease of administration, and completion time. Following pilot testing, we modified the survey to enhance its flow, clarity, and response reliability.^18^, ^19^ The final survey included 21 items and required approximately 5–6 min to complete. The final survey instrument was translated into French and back‐translated into English for accuracy and was offered in both official languages.

### 2.1 Definitions

We defined cannabis use as authorized and nonauthorized use of any medical and non‐medical cannabis product without any distinction between the use of THC or CBD products. Excluded from this survey were pharmaceutically derived nabiximols and synthetic THC analogs, dronabinol and nabilone. Regardless of how or where the cannabis was obtained, the intent had to be for medical purposes; physicians were to exclude considerations for recreational or accidental cannabis exposures.

### 2.2 Geographic regions

Due to the smaller number of physicians in some provinces, we collapsed provinces in Canada into three geographic regions: Western Canada (British Columbia, Alberta, Saskatchewan, Manitoba); Central Canada (Ontario, Québec); and Eastern Canada (Newfoundland and Labrador, Nova Scotia, Prince Edward Island, New Brunswick). Territories, Nunavut, and Yukon were excluded due to the absence of academic pediatric health centers in these regions.

### 2.3 Survey administration and dissemination

The survey was created in Research Electronic Data Capture (REDCap).^20^ We prepared the list of the eligible physicians from the children oncology group website and by contacting the pediatric centers. All eligible participants received a personalized invitation and a link to the survey by email. We sent three reminders at 3‐week intervals to non‐responders. Survey completion was voluntary. Data were automatically entered into a deidentified REDCap database. Upon completing the survey, physicians could opt into a raffle to win one of five $100 e‐gift cards; contact details for this purpose were not linkable to ensure the anonymity of the survey responses.

### 2.4 Statistical analysis and ethics approval

We used frequency distribution to depict the frequency or count of the different variables in the data set. Data analysis was performed using Stata®, Version 15.1 (College Station, TX). The responses to the open‐ended question of participant's perspective of using cannabis for children with cancer were analyzed using the summative content analysis approach.^21^ Two authors (S.O. and J.P.) independently read, analyzed, and coded the free‐text answers to this open‐ended question. The codes were then grouped into thematic categories to map all the qualitative responses. This study was approved by the institution's Health Research Ethics Board.

## 3 RESULTS

The survey was sent to 259 individuals. A total of 122 (47.1%) physicians completed questions about cannabis. Three participants were excluded because they did not meet the study inclusion criteria. The final study population included 119 participants. Overall, 79% were physicians, 21% were fellows, 50.4% were from Central Canada, 40.4% were from Western Canada, and 9.2% were from Eastern Canada.

### 3.1 Education and desired knowledge about medical cannabis

In total, 62.2% of physicians reported completing formal or informal training, most commonly with peer‐reviewed sources (70.5%) and at conferences or workshops (50.4%; Table S1). Many physicians indicated that other forms of training were preferable to conferences or workshops, including webinars or video lectures (64.7%), peer‐reviewed publications (63.9%), or an online portal with collated scientific literature (58.8%). Nearly all physicians (95.8%) wanted to know more about the dosing, side effects, and safety of cannabis. Other areas of desired knowledge included potential therapeutic uses of cannabis (79.8%), different formulations of cannabis (72.3%), and regulations related to the use of cannabis (72.3%; Table S2).

### 3.2 Perspectives on the use of medical cannabis

Most physicians (91.6%) provided care to at least one child with cancer using cannabis for symptom management within the last 6 months. Additionally, more than half (56.8%) had one patient in their clinical practice using cannabis as an adjuvant anticancer agent in the previous 6 months (Table S2). The reported routes of cannabis administration used by children with cancer were oral (88.2%), smoking (31.9%), vaping (21%), sublingual (21%), topical (15.1%), and rectal suppository (8.4%). One‐third of physicians (34.5%) described institutional‐level policies surrounding the use of medical cannabis for pediatric oncology patients in their organizations, 28.4% reported the absence of such policies, and the remainder (37.1%) were unaware of whether or not their institutions had such policies. For patients and caregivers interested in seeking information about the role of cannabis, 57.1% of physicians referred them to other health care professionals knowledgeable about cannabis, and 10.1% reported the use of educational pamphlets provided by licensed producers. Few physicians (8.4%) said that their institution's website had public‐facing cannabis information.

**FIGURE 1:**
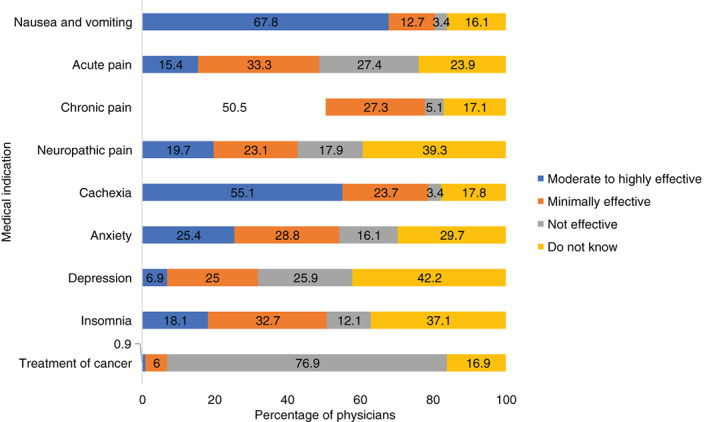
Perceived effectiveness of cannabis for symptom control and treatment of cancer (*N* = 119)

Most physicians (79%) said that patients and caregivers initiated discussions about cannabis; whereas 14% stated that discussions were sometimes started by them and sometimes by families (Table S2). Nearly all (95.8%) physicians identified a potential role for cannabis in pediatric oncology (Table 1), with improvements in chemotherapy‐induced nausea and vomiting (85.7%), chronic pain (72.3%), cachexia/poor appetite (67.2%), anxiety/depression (42.9%) and insomnia (27.7%). Conversely, only four physicians (0.3%) felt that cannabis has a role as an anticancer agent in pediatric oncology. More than half of the physicians perceived cannabis to be moderate to highly effective for managing nausea and vomiting (67.8%), chronic pain (50.5%) and cachexia (55.1%). Cannabis was also perceived to be moderate to highly effective for the treatment of anxiety (25.4%), neuropathic pain (19.7%) and insomnia (18.1%). In contrast, only one physician reported cannabis to be moderately effective as an anticancer agent (Figure 1). Twenty‐two percent of the physicians perceived medical cannabis as usually beneficial for children with cancer receiving palliative care. At the same time, 39.8 and 54.2% reported it to never be helpful in children receiving cancer treatment and childhood cancer survivors, respectively (Table S3). Multiple concerns were identified by physicians related to the use of medical cannabis in children with cancer, with most concerns being uncertain benefits (89.9%), dosing safety (80.7%), and uncertain side effects (75.6%; Table 2).

**TABLE 1:** Potential therapeutic role of cannabis in pediatric oncology reported by physicians (*N* = 119)^a^

Variable	Number	%
Nausea and vomiting	102	85.7
Chronic pain	86	72.3
Cachexia	80	67.2
Anxiety/depression	51	42.9
Insomnia	33	27.7
Acute pain	30	25.2
Cancer	4	3.4
No role	5	4.2
Other^b^	8	6.7

^a^ Not mutually exclusive.

^b^ Lacking evidence/need more data (*n* = 3); placebo (*n* = 1); palliative care only (*n* = 1); individualized or other benefits (*n* = 5).

**TABLE 2:** Concerns related to medical cannabis use in pediatric oncology patients^a^ (*N* = 119)

Variable	Number	%
Uncertain benefits	107	89.9
Dosing safety in children	96	80.7
Uncertain side effects	90	75.6
Uncertainty about quality of products offered by licensed producers	88	74.0
Concern about abuse/misuse	61	51.3
Lack of child‐friendly formulations	43	36.1
Legal implications	41	34.5
Health organization not supportive of medical cannabis in children	12	10.1
Other^b^	5	4.2

^a^ Not mutually exclusive.

^b^ Of the five participants reporting “other” concerns, four provided additional details: *n* = 1 cannabis‐drug interactions; *n* = 1 cost of products and lack of insurance; *n* = 1 concern about long term side effects; *n* = 1 inability to assess subjective side effects).

### 3.3 Perspectives on future research on the use of medical cannabis

**FIGURE 2:**
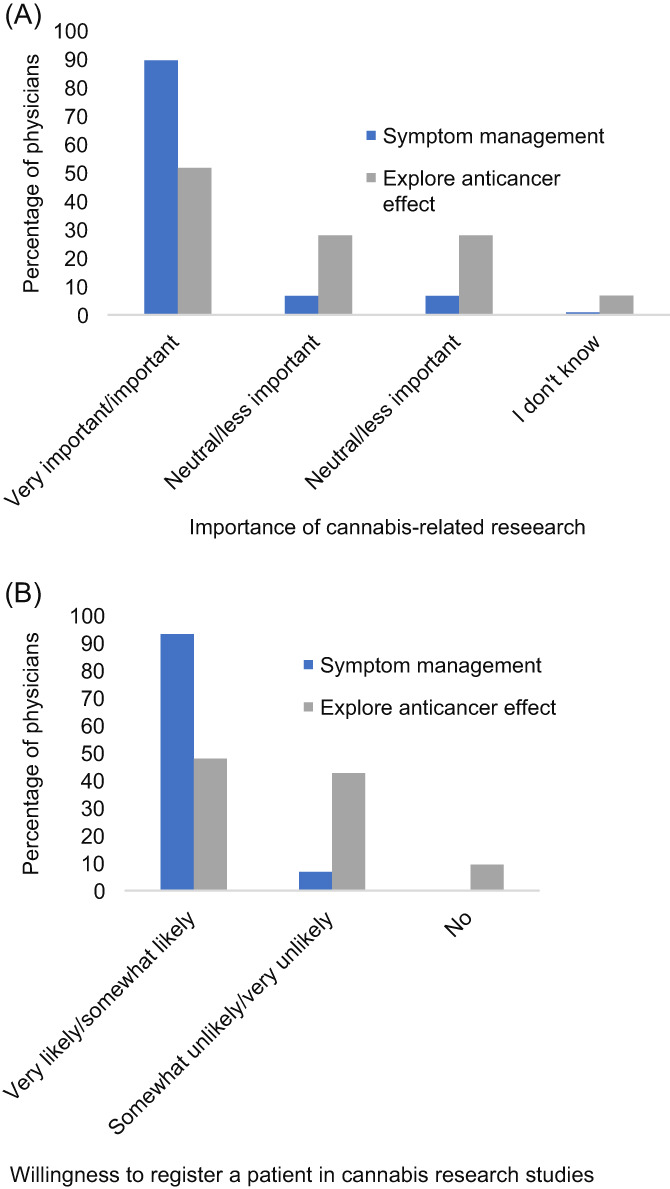
(A) Physicians' perspectives on the importance of conducting studies to explore the efficacy and safety of cannabis for symptom management and as an anticancer agent (*N* = 119); (B) Willingness of physicians to enroll patients in cannabis‐related research studies (*N* = 119)

Almost all physicians (91.5%) considered the conduct of studies investigating the role of cannabis in treating cancer‐ or cancer‐related symptoms to be very important or important, and stated that they would likely enroll their patients in such a study (93.2%) (Figure 2). One‐half of physicians (51.7%) also conveyed that it is crucial to conduct studies to determine the anticancer effects of cannabis; 47.9% of physicians expressed their desire to enroll their patients in such a study (Figure 2).

### 3.4 Analysis of the physicians' qualitative responses

Table 3 describes the common categories that emerged from the analysis of 21 physicians' free‐text responses and relevant quotes. Physicians' responses highlighted caregivers' growing interest in using cannabis as a natural or alternative medicine and strong beliefs in its positive effects. The lack of high‐quality research and the need for robust research on cannabis emerged as common themes. Patient engagement, a nonjudgmental approach to cannabis use, and shared decision making were also considered necessary by the physicians. A few physicians reported concerns about cannabis‐associated harms, the financial burden imposed by cannabis use, and the need for institutional policies.

**TABLE 3:** Categories of physicians' perspectives related to cannabis use in pediatric oncology

Categories	Definition	Example
Lack of high‐quality research	Broader statements about the absence of high‐quality and unbiased studies to inform clinical practice	“Preclinical and clinical studies regarding the safety and efficacy of medical cannabis has been dominated by a company‐driven and sponsored investigator‐driven research ‐ for any other ‘medication’ this would be unusual and highly suspicious.” “There is a marked lack of high‐quality research in its use and most supplies are not very predictable.”
Patients' or Parents' beliefs	General descriptions about the patients' or parents' expectations from the use of cannabis	“Unfortunately, this product has suffered from a ‘desire’ and ‘belief’ for it to work among patients and publications. Parents seem to prefer this, sometimes to standard treatment, because it's ‘natural’.” “In palliative care, families typically wish to start it as a curative, not palliative adjunct, treatment based on certain misrepresentations online about cannabis as a miracle cure.” “Patients are convinced that cannabis will have positive (and sometimes expected to be miraculous) effects and minimal side effects.”
Need for high‐quality studies	Statements about the importance of conducting well‐designed unbiased high‐quality studies	“We need to generate robust scientific data about cannabis so that we can better inform our families' choices and understanding of help during cancer journeys.” “I think that until these things are properly studied, families will keep requesting cannabis or obtaining it from other sources.”
Misinformation from the online content	Descriptions about the inaccurate information related to cannabis use on social media and web	“My main objection to its use in palliative care is that families typically wish to start it as a curative, not palliative adjunct, treatment based on certain misrepresentations online about cannabis as miracle cure.“
Patient engagement and informed decision making	Involving patients in informed decision making and in research studies	“There needs to be an open discussion with patients or their parents regarding the use of cannabis. Physicians should not be judgmental; this way families can trust the health care system and disclose the use of cannabis. Very important to engage parents and families in this research.” “I struggle with being able to counsel patients about something that we don't know much about.”
Policies around use of cannabis	Importance of policies around the use of cannabis	“Institutes or organizations need to have policies in place for or against the use of cannabis in children with cancer.”
Possible harms	Concerns about the harms associated with cannabis use	“I worry patients with a good prognosis will get exposed and potentially lead to unnecessary addiction.”
Financial burden	Financial concerns related to cannabis use	“I think it would be very important to explore the cost/financial implications of cannabis therapy. It's hard to recommend a therapy when the cost is so prohibitive even it's for symptom management.”

## 4 DISCUSSION

In this first Canadian study investigating the perspectives and practices of pediatric oncologists and palliative care physicians regarding the use of cannabis for medical purposes among children with cancer, we found that most physicians provided care to children using medical cannabis, perceived the potential role of cannabis for symptom relief, wanted to learn more about medical cannabis, and considered cannabis‐related research to be crucial.

The number of physicians reporting the use of medical cannabis in their pediatric patients in the last 6 months (91.6% > 1 patient, 19.3% > 5 patients) is significant, given the lack of reliable data on the efficacy of cannabis in pediatric oncology. The frequency of patients using cannabis encountered by physicians in our study aligns with reports from adults with cancer.^22^, ^23^ In a US survey of pediatric oncology health care providers, 30% of providers received more than one request for cannabis in the previous month.^12^ A survey administered by the Canadian Pediatric Surveillance Program (CPSP) in 2017 found that 50% of pediatricians encountered at least one patient who had used cannabis for medical indications in the last 12 months.^24^ Since the proportion of pediatric oncologists or pediatric palliative care physicians completing the CPSP survey is unknown, direct comparison with this study is difficult.

The majority of data on the use of cannabis‐based therapies in children comes from the field of pediatric epilepsy, where CBD and CBD‐enriched whole plant extracts containing minor amounts of THC in a 1:20 THC: CBD ratio have been found to be beneficial in reducing the frequency of seizures in children with Dravet syndrome, Lennox–Gastaut syndrome or tuberous sclerosis.^5^, ^6^, ^7^ In pediatric oncology, three cross‐sectional studies enrolling 50, 27, and 14 participants have explored the experiences of children or their caregivers of using medical cannabis.^9^, ^25^, ^26^ The majority of the children or their caregivers (80–100%) in these studies reported improvements in nausea and vomiting, reduced appetite, pain, insomnia and anxiety.^9^, ^25^, ^26^ Only a small proportion (14%) reported side effects related to cannabis use, such as throat and abdomen pain.^9^ In another qualitative study, adolescents and young adults with cancer, and their parents, endorsed cannabis to manage nausea, pain, and anorexia after weighing the risk of potential physiological and psychological side effects of cannabis.^27^ The majority of physicians in our study also believed that cannabis has a role in symptom control, especially among children receiving palliative care. The physicians' perspectives likely symbolize the positive benefits observed in their clinical practice or their awareness of the emerging data on the benefits of cannabis for symptom control among children with cancer, especially in a palliative care setting where the risks associated with cannabis are low.^28^, ^29^, ^30^

Although the above‐mentioned observational studies signal the potential benefits of cannabis use in pediatric oncology, no robust clinical trials have identified the appropriate dosing, THC/CBD ratio, efficacy, and long‐term safety of cannabis products for symptom control in pediatric oncology. Not surprisingly, the vast majority of physicians in our study considered cannabis‐related research for symptom control (91.5%) in pediatric oncology to be very important, and nearly all (93.2%) expressed their desire to enroll patients in clinical trials. These data underscore the pressing need for expedited, well‐designed cannabis clinical trials to meet the demand of patients, families, and healthcare professionals. In the meantime, formal surveillance programs to collate observational data on cannabis product selection, dosing, safety, and benefits are needed to inform families, clinicians, and future research.

In our study, smoking and vaping were identified as the second most common route for cannabis administration, perhaps due to the rapid onset of action of cannabis consumed by these modes of administration. Smoking and vaping present an issue with accurate dosing, unlike oral cannabis oil dosing, where an exact dose is consumed. Smoking can also increase the short‐ and long‐term pulmonary complications among these patients.^31^, ^32^ Although the pulmonary complications associated with vaping are fewer than smoking, more studies are needed to evaluate this risk in detail.^33^, ^34^, ^35^ Recent pilot studies have demonstrated the safety and efficacy of selective‐dose cannabis inhalers in managing chronic pain in adults. Whether such cannabis delivery modes are safe and efficacious among pediatric oncology patients desiring the immediate effects of cannabis should be the focus of future research.^34^, ^36^ Cannabis use as a rectal suppository, as reported by some physicians, is concerning because rectal manipulation in neutropenic patients can increase the risk of systemic bacterial infection by promoting bacterial translocation.^37^, ^38^ It is essential that physicians advise all patients and their caregivers expressing interest in using cannabis‐based products on these additional route‐of‐administration‐dependent risks.

Few physicians in our study reported the existence of policies for cannabis use in their institutions. Some physicians communicated using information pamphlets developed by licensed producers for their patients, which may provide biased, incomplete, unverified information, and lack supportive resources for additional information.^39^ As the interest in cannabis use increases over time, cancer organizations and pediatric healthcare institutions need to educate physicians, develop policies for in‐hospital use and develop a robust framework for providing accurate information to cancer patients and their families. Meaningful engagement and shared decision making between physicians and families can influence public opinions and mitigate the uptake of false and unvalidated information from nonmedical resources and social media.^39^ More importantly, future efforts must address the specific regulatory barriers to accessing quality cannabis products to be used in research studies exploring the beneficial and harmful effects of cannabis in pediatric oncology patients.

Some of our findings contrast the results of a recently conducted survey of 288 nurses, nurse practitioners, physicians, and physician assistants in the United States regarding cannabis use in pediatric oncology.^12^ One‐third of the participants in this study indicated their approval for the use of cannabis as an anticancer agent^12^ compared to only 3.4% of Canadian physician respondents in our survey. These diverging findings may be attributable to the inclusion of a broader population of clinicians in the US survey, who might have had a varied perspective on cannabis use.

Our findings must be interpreted in light of some limitations. Our survey centered on any cannabis product as physicians are often unaware of the precise contents of the products used by their pediatric oncology patients. Therefore, we did not capture perspectives specific to the use of THC or CBD. Concerns related to the psychoactive properties of THC and its effects on brain development, especially in children, might have shaped some of the responses of the physicians. Future research studies should explore healthcare providers' perspectives on THC and CBD‐dominant formulations in this population. To keep the survey responses deidentified for a smaller number of physicians, we could not collect more detailed demographic and personal variables that might have influenced physicians' responses. Additionally, the actual number of patients using cannabis might have been overestimated due to pediatric oncologists' shared clinical practices or underestimated because of some patients' under‐reporting of cannabis use in clinical practice. Furthermore, selection bias with physicians who do not have cannabis experience or do not believe cannabis plays a role in pediatric oncology choosing not to participate potentially limits the generalizability of our findings.

Despite these limitations, our study is the first to provide data on the perspectives of Canadian pediatric oncologists and palliative care physicians regarding cannabis use in children with a current or previous cancer diagnosis. Several important implications from our findings include an urgent call for research and the development of clinical practice guidelines to support families and health care providers advising on the use of cannabis products in pediatric oncology. Funding agencies would be wise to provide direct funding opportunities for cannabis research in cancer, particularly among pediatric oncology populations where interest and use are rapidly outpacing the generation of rigorous evidence on dosing, efficacy, and safety. Cancer organizations and pediatric healthcare institutions must work with patients and caregivers to co‐develop an evidence‐based framework to provide verified information for the medicinal use of cannabis.

## CONFLICT OF INTEREST

The authors declare no conflict of interest.

## AUTHOR CONTRIBUTIONS

*Conception and Design*, S.O., M.I.V., L.E.K., J.P., A,R., S.R.R., B.C., H.S., K.D., P.A., L.B.; *Collection and Assembly of Data*, S.O., J.P., M.I.V., L.E.K.; *Data Analysis and Interpretation*, All authors; *Manuscript Writing*, All authors; *Final Approval of Manuscript*, All authors; *Accountable for All Aspects of the Work*, All authors.

## ETHICAL STATEMENT

This study was approved by the University of Manitoba Health Research Ethics Board (REB Number: HS23152).

## CONSENT

This was a self‐administered, voluntary survey. Thus, by completing the questionnaire, the participant gave us an “implied consent.”

## Supporting information


**Appendix S1**: Supporting information

## Data Availability

The deidentified individual participant data supporting the findings of this study will be made available upon publication to researchers providing a methodologically sound proposal for using data. The data can be requested from the corresponding author. The data are not publicly available due to privacy or ethical restrictions.
