# Supplementary material for: Perspectives of pediatric oncologists and palliative care physicians on the therapeutic use of cannabis in children with cancer
Source: Cancer Rep (Hoboken). 2021 Oct 21;5(9):e1551. doi: 10.1002/cnr2.1551 (PMC9458513; doi:10.1002/cnr2.1551)
Supplement: Supplementary file 1 — Appendix S1: Supporting information [file CNR2-5-e1551-s001.docx]

**Perspectives of Pediatric Oncologists and Palliative Care Physicians on the Therapeutic Use of Cannabis in Children with Cancer**

***Supporting information***

**Table of contents**

- **Supporting Table 1. Cannabis-related training of physicians (N=119)**
- **Supporting Table 2. Use of medical cannabis in pediatric oncology (N=119)**
- **Supporting Table 3. Reported benefits of cannabis for select subgroups of patients in pediatric oncology (N=118)**

**Supporting Table 1. Knowledge of physicians regarding cannabis use in pediatric oncology (N=119)**

| Variable | | n | % |
| --- | --- | --- | --- |
| Formal training for use of cannabis to treat medical conditions | |  |  |
|  | No | 45 | 37.8 |
|  | Yes | 74 | 62.2 |
| Sources of training^a^ | |  |  |
|  | Peer-reviewed sources | 84 | 70.5 |
|  | Non-peer-reviewed sources | 32 | 26.9 |
|  | Government, or medically reviewed online programs | 25 | 21.0 |
|  | Conferences/workshops | 60 | 50.4 |
|  | Licensed producers | 15 | 12.6 |
|  | Community-based dispensaries | 4 | 2.4 |
|  | Patients and families | 33 | 27.7 |
|  | I do not access any information on cannabis | 11 | 9.2 |
|  | Other^a,b^ | 16 | 13.5 |
| Desired knowledge about cannabis^a^ | |  |  |
|  | Potential therapeutic uses | 95 | 79.8 |
|  | Dosing, side effects and safety | 114 | 95.8 |
|  | Mechanisms of action | 67 | 56.3 |
|  | Different formulations | 86 | 72.3 |
|  | Laws and regulations related to use | 86 | 72.4 |
|  | Other^a,c^ | 6 | 5.0 |
| Preferred method of cannabis education^a^ | |  |  |
|  | Webinar or video lecture | 77 | 64.7 |
|  | Peer-reviewed publications | 76 | 63.9 |
|  | Conferences/workshops | 55 | 46.2 |
|  | Online portal with collated scientific literature | 70 | 58.8 |
|  | Other^a,d^ | 3 | 2.5 |
|  |  |  |  |
| ^a^Not mutually exclusive | |  |  |
| ^b^CADTH, government websites, online resources (n=3); colleagues, mentors, prescribers (n=14) | | | |
| ^c^Includes drug interactions (n=2); mode of administration (n=1); potential for neurotoxicity (n=1), | | | |
| long term effects on development/cognition (n=1); different cannabinoids for different treatment (n=1) | | | |
| ^d^Includes collaborations with basic science research (n=1); colleagues, mentors (n=1); presentations (n=1) | | | |

| **Supporting Table 2. Use of medical cannabis in pediatric oncology (N=119)** | |  |  |
| --- | --- | --- | --- |
|  |  |  |  |
| Variable | | n | % |
| Initiate conversation | |  |  |
|  | MD, usually | 0 | 0.0 |
|  | Patient and family, usually | 94 | 79.0 |
|  | Sometimes MD, sometimes patient and family | 17 | 14.3 |
|  | Other healthcare professionals | 3 | 2.5 |
|  | Not discussed | 5 | 4.2 |
| Number of patients known to have used cannabis for symptom management^a^ | | | |
|  | None | 10 | 8.4 |
|  | 1-5 | 73 | 61.3 |
|  | 6-10 | 23 | 19.3 |
|  | 11-15 | 8 | 6.7 |
|  | 16-20 | 2 | 1.7 |
|  | >20 | 3 | 2.5 |
| Number of patients known to have used cannabis as an anticancer agent^a,b^ | | | |
|  | None | 51 | 43.2 |
|  | 1-5 | 57 | 48.3 |
|  | 6-10 | 8 | 6.7 |
|  | 11-15 | 1 | 0.9 |
|  | 16-20 | 0 | 0.0 |
|  | >20 | 1 | 0.9 |
|  |  |  |  |
| ^a^Includes both authorized and non-authorized use, in the past 6 months  ^b^ N=118 | | | |

| **Supporting Table 3. Reported benefits of cannabis for select subgroups of patients in pediatric oncology (N=118)**   \| **Variable** \| **Number** \| **%** \| \| --- \| --- \| --- \| \| Children receiving curative treatment  Never or rarely beneficial  Sometimes beneficial  Usually beneficial  Always beneficial  I don’t know \| 47  58  1  0  12 \| 39.8  49.1  0.9  0  10.2 \| \| Children receiving palliative treatment  Never or rarely beneficial  Sometimes beneficial  Usually beneficial  Always beneficial  I don’t know \| 7  80  26  1  4 \| 5.9  67.8  22.0  0.9  3.4 \| \| Childhood cancer survivors  Never or rarely beneficial  Sometimes beneficial  Usually beneficial  Always beneficial  I don’t know \| 64  25  2  0  27 \| 54.2  21.2  1.7  0  22.9 \| |
| --- | --- | --- | --- | --- | --- | --- | --- | --- | --- | --- | --- | --- |
